# PULSE: A Fast Portable Unit for Lab-on-Site Electrochemistry

**DOI:** 10.3390/s25030762

**Published:** 2025-01-27

**Authors:** Cláudia Ferreira, Fiona Barry, Miomir Todorović, Patrick Sugrue, Sofia Rodrigues Teixeira, Paul Galvin

**Affiliations:** Tyndall National Institute, University College Cork, T12 R5CP Cork, Ireland; fiona.barry@tyndall.ie (F.B.); miomir.todorovic@tyndall.ie (M.T.); patrick.sugrue@tyndall.ie (P.S.); sofia.teixeira@tyndall.ie (S.R.T.); paul.galvin@tyndall.ie (P.G.)

**Keywords:** electronics, electrochemistry, sensors, potentiostat, firmware, hardware

## Abstract

This study aims to develop and validate a novel fast-detection electrochemical sensing platform to enhance portable electrochemical sensor solutions. The research focuses on optimising analogue front-end circuits, developing data analysis algorithms, and validating the device through experiments to enhance measurement accuracy and detection speed, enabling on-site measurements across diverse applications. This work successfully designed a Portable Unit for Lab-on-Site Electrochemistry (PULSE) system with dimensions of (78×100×2) ${\mathrm{mm}}^{3}$. The device’s implementation was complemented by robust firmware that performed desired electrochemical measurements, including open circuit potentiometry (OCP), chronoamperometry (CA), and cyclic voltammetry (CV). To assess its reliability, the PULSE was benchmarked against a well-established benchtop potentiostat. The results obtained highlight the system’s rapid sensing capabilities, achieving pH detection in 2 s and performing CA in 20 s. The pH calibration curve exhibited Nernstian behaviour with an accuracy of 97.58%. A correlation analysis comparing the calibration curve datasets across all electrochemical techniques from both systems revealed high correlation coefficients (>0.99), confirming the strong agreement between the two systems.

## 1. Introduction

In recent years, there has been a growing demand for portable and versatile electrochemical sensing platforms that provide accurate and real-time measurements for various applications [1,2,3]. These platforms enable on-site analysis, facilitating rapid decision making and reducing the need for laboratory-based equipment, thereby lowering associated costs [4]. Such platforms hold immense potential for a wide range of applications, including food safety [5], drug detection [2], diagnosis of virus such as SARS-CoV-2 [6], and environmental monitoring [7]. However, the design, optimisation, and validation of comprehensive portable electrochemical devices face several limitations, holding back their widespread adoption. This study aimed to address the need for a fully optimised, portable electrochemical sensing platform capable of real-time, multi-parametric measurements.

Existing devices often lack the required adaptability, configurability, and affordability necessary to meet the diverse needs of researchers and users [4,8,9,10]. A review by Umapathi, R., et al. highlighted the urgency of developing stable, intelligent, and adaptable platforms for detecting food contaminants, emphasising the need for robust electrochemical sensing technologies [4]. Another review on portable devices for drug detection underlined the need for hardware and software improvements to enhance signal detection [8]. A study conducted by Scott, A., et al. demonstrated the development of a portable device with constraints in adjustability, operational range (approximately 10 μA), and communication capabilities [10]. A study focused on validating a potentiostat for the detection of periodontal disease demonstrated its ability to perform cyclic voltammetry (CV), and amperometry [11]. However, the calibration curves presented $R^{2}$ values below 0.95, which may not be sufficient to replace benchtop potentiostats, indicating room for improvement. Additionally, a review of electrochemical biosensors for virus detection identified signal processing as a critical area for improvement; advancements in this aspect could result in more accurate signal acquisition [12].

This study addressed the limitations of portable electrochemical sensing platforms by developing a comprehensive solution: the PULSE (Portable Unit for Lab-on-Site Electrochemistry). The aim of this study was to provide accurate, real-time measurements, including CV, chronoamperometry (CA), and open circuit potentiometry (OCP), in diverse applications and environments. The approach involved optimising analogue front-end (AFE) circuits to enhance tailored applications, developing data analysis algorithms for accurate signal identification, and conducting experimental validation. This work highlighted the integration and design of key system components, resulting in a proof-of-concept system for analyses requiring multi-parametric capabilities.

## 2. Materials and Methods

### 2.1. Hardware Development

**Figure 1:**
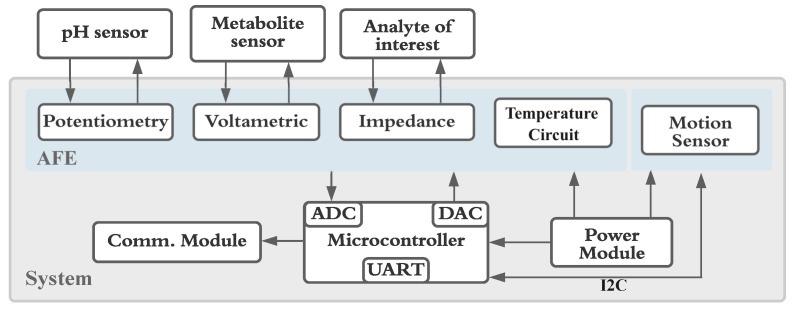
Overall system block diagram, including AFE (analogue front-end), ADC (analogue-to-digital converter), DAC (digital-to-analogue converter), UART (universal asynchronous receiver transmitter), and I2C (inter-integrated circuit).

The electronic system was designed as a modular block system, as shown in Figure 1, allowing for flexibility and redesign to meet specific requirements. The measurement system supported a standard 3-electrode connection or 2-electrode configuration sensors, along with temperature and acceleration measurement capabilities. Acting as an intermediary within the system, the AFE, to which the sensors are connected, processed the sensor signals, conditioned them for conversion, and ensured compatibility with subsequent system stages [13]. The device was powered through a USB cable connected to a laptop, which supplied 5 V and enabled communication with the user device through a universal asynchronous receiver transmitter to a universal serial bus (USART-to-USB) converter. Wireless communication at 433 MHz was supported by an antenna (0433AT62A0020E from Johanson Technology Inc., Camarillo, CA, USA), which benefited from regulatory approval for industrial, scientific, and medical (ISM) applications, and its properties in underwater environments [14,15]. The printed circuit board (PCB) design was meticulously executed, selecting compact component sizes and arranging multiple components in blocks to occupy a reasonable area suitable for a portable device. An application (APP) was developed using Qt software 5.15 (Qt, Helsinki, Finland) to enable device control, supporting Linux, OS, Windows, Android, iOS, and other operating systems. The implementation encompassed the development of a comprehensive communication protocol, with an extensive command list and the integration of a graphical user interface (GUI) within the APP to facilitate the presentation of measurement results.

To ensure reliable measurements, the 12-bit internal analogue-to-digital converter (ADC) of the Microcontroller Unit (MCU) (STM32F372R8T6, STMicroelectronics, Geneva, Switzerland) was calibrated using a benchtop voltage generator for voltage sweeping. The voltage was recorded via a high-precision multimeter and the PULSE’s serial port, followed by linear regression analysis. For precise voltage control, an external digital-to-analogue converter (DAC) (DAC8568ICPW by Texas Instruments, Dallas, TX, USA) was included and calibrated to establish the relationship between the digital input code and the actual output voltage. All the calibration equations were embedded within the APP.

#### 2.1.1. Voltammetric AFE

**Figure 2:**
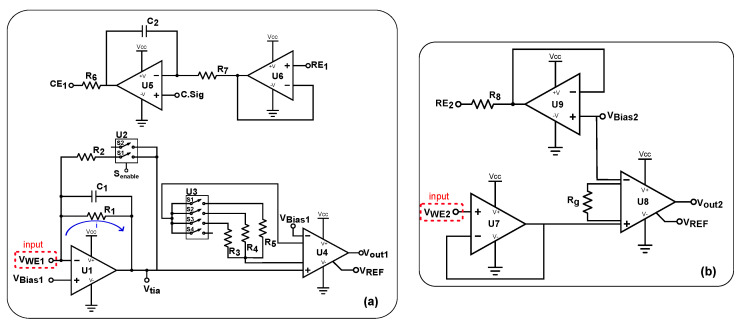
Simplified schematic circuit design for (**a**) voltammetric measurements and (**b**) potentiometric measurements.

The design of a voltammetric circuit was crucial for ensuring accurate and undistorted measurements in voltammetry [16]. The implemented circuit is depicted in Figure 2a. As described in [17], three essential components form a potentiostat: the control amplifier (U5—ADA4807 from Analog Devices Inc., Wilmington, MA, USA) [18], the electrometer (U6—LTC6081 from Analog Devices Inc.) [19], and the current–voltage converter or trans-impedance amplifier (TIA) (U1—LTC6081 from Analog Devices Inc.) [19,20]. Additional details are provided in Appendix A.

The control amplifier (U5) was used to drive large capacitive loads in the electrochemical cell, while U6 functioned as a buffer. Together, C2 (470 pF) and R7 (10 kΩ) formed a pole that alters the unity-gain frequency, thereby influencing the phase margin and stabilizing the system. The resistor R6 (10 Ω) prevented ringing and distortion by providing out-of-loop compensation. Positioned at the output of U5, R6 acted as an isolation resistor between the op-amp output and the load, ensuring that it remained outside the feedback loop.

To enhance the system’s versatility, this study integrated an instrumentation amplifier (U4—INA333 from Texas Instruments) with a variable gain, which was connected to a complementary metal oxide semiconductor (CMOS) analogue multiplexer (U3—ADG704 from Analog Devices Inc.) featuring a low channel resistance of 2.5 Ω. This multiplexer was responsible for selecting the gain resistor ($R_{g}$). The TIA (U1) generated an output voltage proportional to the input current from the working electrode (WE). This component included a feedback loop composed of two branches. The first branch was fixed, consisting of R1 in parallel with C1, while the second branch, which could be activated as needed by U2, consisted of R2. The R2—U2 circuit served as an automatic gain control (AGC) mechanism for the TIA, regulated by the MCU signal ($S_{enable}$), which enabled the selection of the appropriate branch in U2. Once the measured signal exceeded a certain threshold, R2 could be activated, allowing measurements across a wider range. This ensured that the TIA remained compliant and maintained a linear response without drifting. Using the fundamental principles of virtual ground in operational amplifiers (OpAmps) operating in linear mode, $V_{Bias1}$ was equivalent to the negative input signal of the TIA ($V_{WE1}$). Consequently, the current could be expressed as:

(1) $$I=\frac{V_{tia}-V_{Bias1}}{R_{f}}$$

where $R_{f}$ is the value of the feedback resistor of the TIA. Additionally, this equation was used for a comprehensive study of the operational range of this circuitry.

Furthermore, the noise of the voltammetric and potentiometric AFE was evaluated. The transimpedance circuit comprised a feedback resistor ($R_{f}$) of 9.9 kΩ and a feedback capacitor ($C_{f}$) of 10 nF. The AFE circuitry was connected to a dummy cell from Metrohm Autolab (Cheshire, UK), which mimicked the properties of an electrochemical cell. The input voltage was varied to obtain the full signal range from 0 V to 3.3 V, and the noise was assessed by analysing the peak-to-peak differences in the time-domain recorded data. These differences were then used to calculate the signal-to-noise ratio (SNR). Additionally, power consumption was measured using a Power Profiler Kit II (PPK2) (Nordic Semiconductor, Trondheim, Norway) to monitor the current driven by the PULSE. The PPK2 operated in source meter mode, measuring the current while providing power to the device (3.5 V). The power was calculated as the product of the supply voltage and the measured current.

#### 2.1.2. Potentiometric AFE

The developed circuit, shown in Figure 2b, was an adaptation of the 2-electrode methodology previously reported in [21]. This circuit was designed to measure the voltage drop between the reference electrode (RE) and the WE. To achieve this, the voltage signal was first buffered by U7 (LMP7721 from Texas Instruments), which was connected to the WE. The LMP7721 was chosen for its low input bias current of 20 fA at 25 °C, providing high input impedance and ensuring no losses or alterations in the signal [22]. The instrumentation amplifier U8 (INA333 from Texas Instruments) can be considered high impedance with a low input bias current of typically 70 pA. However, this input bias current can negatively affect the pH sensor’s stability and durability. For pH sensing, an input bias current lower than 1 pA is desirable, as identified in Section E. The RE is maintained at a constant voltage using the U9 (ADA4807 from Analog Devices Inc.) amplifier, which can be enabled or disabled, minimizing interference with other sensors. Applying the same principle as the previous circuit, Equation (2) was derived to calculate the output voltage ($V_{out2}$).

(2) $$V_{out2}=V_{ref}+G\times \left(V_{WE2}-V_{Bias2}\right)$$

The voltage difference between the WE and RE was measured by calculating $V_{WE2}-V_{RE_{2}}$. These equations facilitated a comprehensive study of the operational range of this AFE. Additionally, the circuit was tested for pH measurements to assess its ability to capture pH changes, including detection speed. The pH measurement capabilities are relevant for applications in environmental monitoring [23], biomedical research [24], and industrial processes [25].

### 2.2. Firmware Development

**Figure 3:**
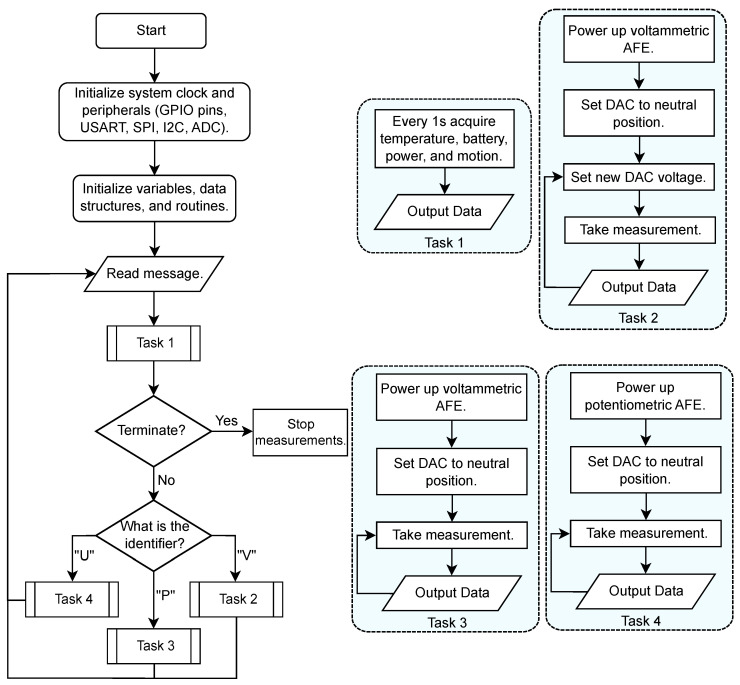
Overview flowchart of the FW procedure, with Task 1 for continuous measurements after the start signal and Tasks 2–4 summarizing the FSMs for CV, CA, and OCP measurements, respectively.

The flowchart in Figure 3 outlines the firmware (FW) development for the MCU [26]. Once the start signal was received, periodic data acquisition began, measuring temperature, battery level, motion, and power data at 1 s intervals. The collected data were subsequently outputted for analysis. Simultaneously, the system processed input data from the APP and checked for termination conditions. If no stop signal was received, data acquisition continued for specific measurements. A finite-state machine (FSM) approach for CA, CV, and OCP was implemented, executing a new state every 100 ms, as illustrated in Figure 3 Tasks 2, 3, and 4, respectively. Communication with the APP was established via a serial port operating at 115,200 bauds, with a robust serial protocol for reliable communication. The protocol structure included a start index, a channel identifier, data in hexadecimal format, and a stop index. Acquired data were then outputted and analysed at the APP level. This process continued until termination conditions were met. Additionally, an exclusive OR (XOR) checksum process ensured message integrity and error handling [27].

In addition to the FW development process, the implemented software supported three key electrochemical measurements: CV, CA, and OCP. Depending on the technique, $V_{ref}$, $V_{Bias1}$, $V_{Bias2}$, and C.Sig were set to 0 V or 1.65 V. These voltage settings, chosen at *Vcc*/2 (with *Vcc* = 3.3 V), enabled both polarity measurements on the electrochemical cell, biasing the voltage at the signal range midpoint. For further details on each method, see Appendix B.

Integrating these techniques into the FW enabled versatile electrochemical analysis. CA provided time-dependent current data for real-time monitoring, CV investigated redox reactions and kinetics for chemical analysis, and OCP provided time-dependent voltage for dynamic monitoring. This study validated each method, ensuring electrochemical reliability.

### 2.3. Validation

**Figure 4:**
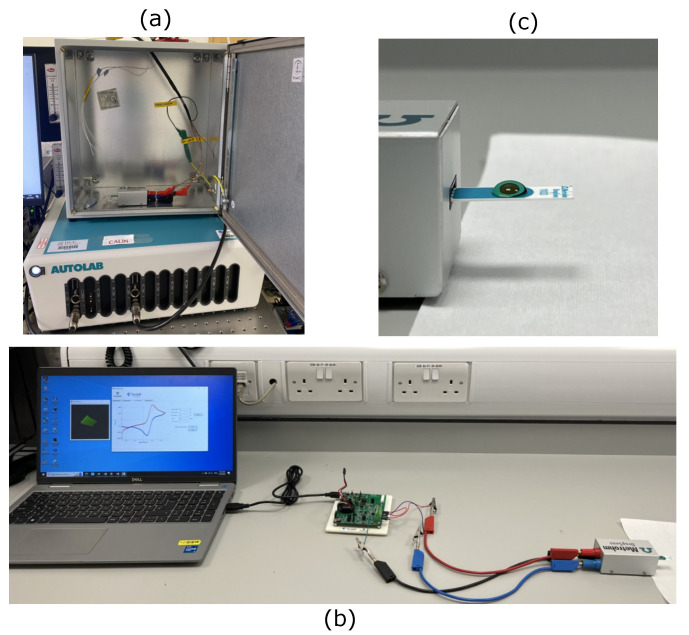
Validation experiment setup in the laboratory at Tyndall National Institute facilities using (**a**) a commercial benchtop system (Metrohm Multi Autolab M101), (**b**) PULSE and the developed APP, and (**c**) screen-printed electrodes obtained from DropSens (DRP—110GPH).

To validate the PULSE system, the device was benchmarked against a commercial benchtop potentiostat—Metrohm Multi Autolab M101 (Utrecht, The Netherlands)—available at Tyndall National Institute facilities (Figure 4a), performing CA, CV, and OCP. The electrochemical measurements were carried out using carbon screen-printed electrodes (SPEs) obtained from DropSens (DRP–110GPH) (Figure 4c). These SPEs consisted of a carbon WE (4 mm diameter), a carbon counter electrode (CE), and a silver/silver chloride (Ag/AgCl) RE. CA, CV, and OCP were performed in triplicate.

Potassium hexacyanoferrate III ($K_{3}$[Fe(CN${)}_{6}$]), potassium hexacyanoferrate II ($K_{4}$-[Fe(CN${)}_{6}]$), phosphate-buffered saline (PBS), iridium (IV) chloride (${\mathrm{IrCl}}_{4}\cdot H_{2}O$), hydrogen peroxide ($H_{2}O_{2}$), oxalic acid ($C_{2}H_{2}O_{4}$), and sodium carbonate (${\mathrm{Na}}_{2}{\mathrm{CO}}_{3}$) were obtained from Sigma Aldrich (Gillingham, UK). PBS solutions (10 mM) were prepared by dissolving one PBS tablet (pH 7.4) in 200 mL of deionized water. pH measurements were carried out in PBS solution, with sodium hydroxide (NaOH) or hydrochloric acid (HCl) added to adjust the pH as required. Potassium hexacyanoferrate III and potassium hexacyanoferrate II solutions were prepared in PBS. All solutions were prepared using deionized Milli-Q water (resistivity of 18.2 MΩcm).

Prior to pH analysis using OCP, the WEs were functionalised with an iridium oxide (IrOx) sensing layer, using the Yamanaka method [28]. IV chloride hydrate (${\mathrm{IrCl}}_{4}\cdot H_{2}O$) was dissolved in deionized water and stirred for 30 min. Then, 1 mL of aqueous 30% $H_{2}O_{2}$ was added to the solution and stirred for 30 min. This was combined with 0.5 g of oxalic acid ($C_{2}H_{2}O_{4}$) and stirred for 30 min. Sodium carbonate (${\mathrm{Na}}_{2}{\mathrm{CO}}_{3}$) was added to adjust the pH to ∼10. For the electrochemical deposition of the IrOx solution, 80 CV scans were performed with a potential range from −0.7 V to 0.6 V at a scan rate of 10 mV/s.

For CV measurements using the PULSE system, the setup used is illustrated in Figure 4b. The voltammetric circuitry was configured with a gain of 1, $R_{f}$ of 9.9 kΩ, and $C_{f}$ of 10 nF. The potentiometric circuitry for pH measurements used a gain of 1. The tests were conducted without the use of a Faraday cage or other interference reduction methods to replicate real-world scenarios. The collected data were processed, and regression models were generated for the benchtop potentiostat and the PULSE system datasets.

## 3. Results

### 3.1. Hardware

**Figure 5:**
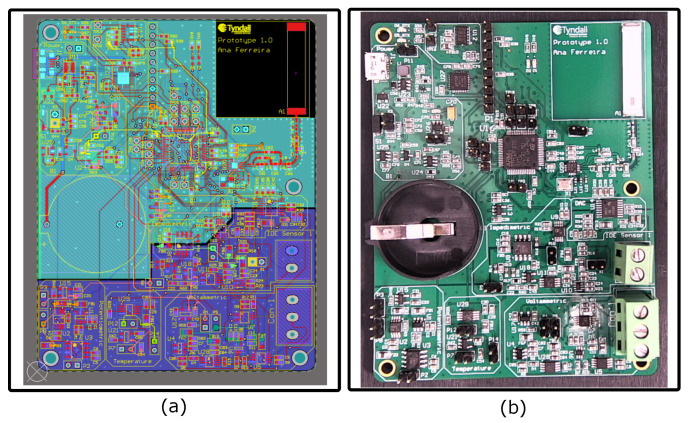
Printed circuit board: (**a**) Altium’s 2D PCB routing interface and (**b**) fabricated PCB.

The PCB layout (Figure 5a) maximised the utilisation of the top layer for ease of testing and repairs, while ensuring a robust ground return. The routing used a 0.16 μm trace width for cost-effectiveness, rerouting away from passive components. Additional ground vias were introduced to reduce noise, with unrestricted multi-layer routing. Polygon pours were used to ensure signal integrity and structural prevention. Additionally, capacitors were carefully selected and positioned for power supply decoupling and filtering [29].

The fabricated PCB (Figure 5b) measured approximately (78×100×2) ${\mathrm{mm}}^{3}$ and weighed 53 g, highlighting its compact and lightweight design, which was crucial for ensuring the device’s portability and suitability for on-site applications. The PCB featured a coin cell battery holder with a diameter of 24 mm, a micro USB-B connector, an antenna, and connectors for both 3-electrode and 2-electrode configurations. This system successfully provided a solid foundation for the subsequent stages of electronic system development and characterisation.

From the calibration of the ADCs, Equations (3) and (4) were derived for the voltammetric and potentiometric AFEs, respectively. These equations represent the linear relationship between real-world voltage values ($V_{real}$) and serial port voltage values ($V_{serial}$), both with $R^{2}$ = 0.999.

(3) $$V_{real}=1.003919\times V_{serial}+0.037731$$

(4) $$V_{real}=1.004436\times V_{serial}-0.0527213$$

Similarly, the calibration equation of the DAC, representing the linear ($R^{2}$ = 0.999) relationship between the digital input code ($D_{in}$) and the actual output voltage ($V_{out}$), is expressed as follows:

(5) $$V_{out}=0.000076\times D_{in}+0.005498$$

### 3.2. Characterisation

#### 3.2.1. Operational Range

The results of the voltage range analysis for the voltammetric and potentiometric AFEs are presented in Table 1 and Table 2, respectively. The operational range of the voltammetric circuit was characterised by current (*I*) for different gain and feedback resistor values. The potentiometric circuitry was characterised by the input voltage of the WE ($V_{WE2}$) and the sensed voltage ($V_{out2}$) between WE and RE. The TIA’s output voltage ranged from 0.1 V to 3.2 V, and the maximum output voltage of the circuits varied from 0.05 V to 3.25 V. These operational ranges are calculated for multiple INA333 gains (1, 2, 10, 100), with varying $R_{f}$ values for the voltammetric AFE. To calculate the values of $V_{tia}$, Equation (A1) was rearranged as

(6) $$V_{tia}=\frac{V_{out1}-V_{ref}}{G}+V_{Bias1}$$

In contrast, the values of $V_{WE2}$ were calculated by rearranging Equation (2) as follows:

(7) $$V_{WE2}=\frac{V_{out2}-V_{ref}}{G}+V_{Bias2}$$

By applying Equation (1), the voltammetric circuit exhibited a broad operational range, as detailed in Table 1. This range spanned from microamperes to nanoamperes, demonstrating its capability. The circuit achieved lower currents compared with other devices [10]. Users could easily adjust gain and $R_{f}$ values through the APP, enabling customisation for specific electrochemical analysis requirements in terms of sensitivity and dynamic range. This flexibility optimised the system for multiple electrochemical applications. Additionally, the ability to modify these parameters via the GUI enhanced the system’s versatility and adaptability to a wide range of experimental conditions.

#### 3.2.2. Noise Levels

The noise levels of the voltammetric and potentiometric AFE circuits were assessed, which demonstrated SNR levels ranging from 25.02 dB to 33.08 dB and 28.06 dB to 33.13 dB, respectively. These measurements were taken across the entire voltage range, indicating the performance and reliability of the circuits under various operational conditions.

#### 3.2.3. Power Consumption

The power consumption of the system was recorded as 81 mW in the idle state. During different electrochemical techniques, the power consumption increased to 87.2 mW for CV, 90.4 mW for CA, and 89.9 mW for OCP measurements. These values provided insights into the energy demands of these specific electrochemical techniques compared with the idle state.

### 3.3. Cyclic Voltammetry

**Figure 6:**
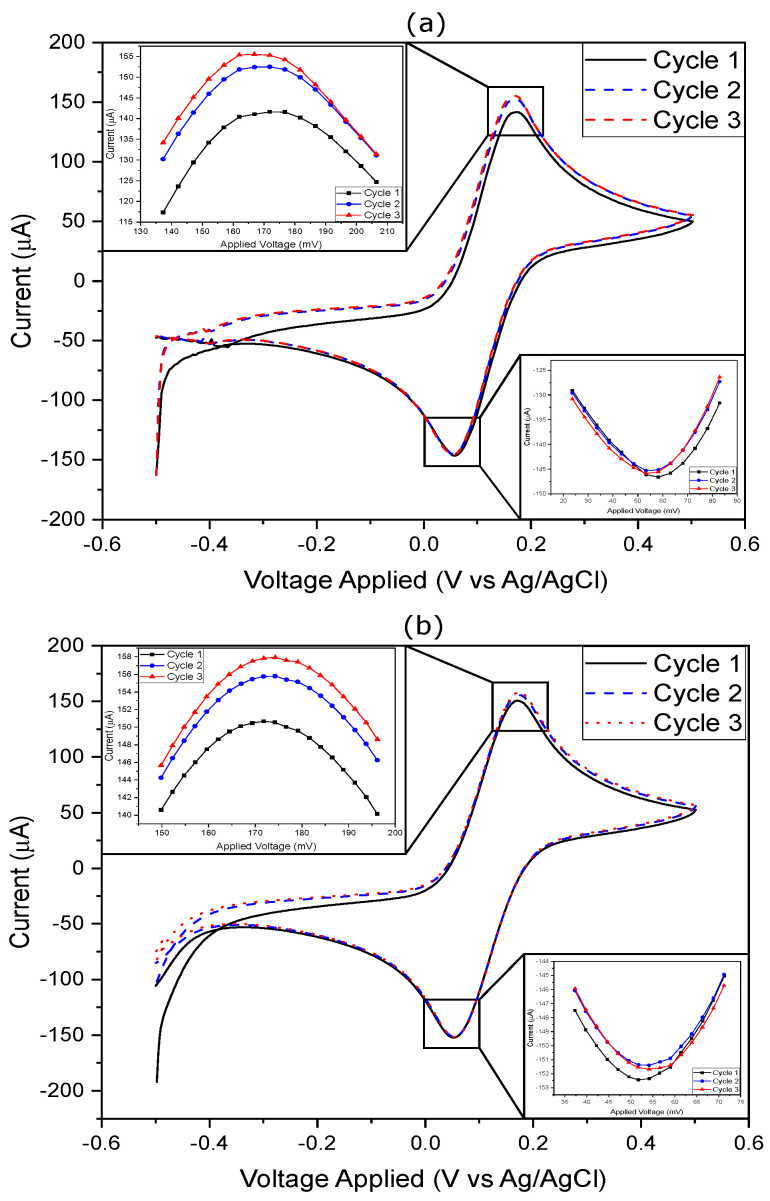
CV results using (**a**) the PULSE system and (**b**) the benchtop potentiostat, where the potential was swept from −0.5 V to 0.5 V at 50 mV/s with a 5 mM concentration of ${\left[\mathrm{Fe}{\left(\mathrm{CN}\right)}_{6}\right]}^{3-}/{\left[\mathrm{Fe}{\left(\mathrm{CN}\right)}_{6}\right]}^{4-}$.

The results obtained from performing CV using the benchtop potentiostat and the PULSE system to detect 5 mM concentration of ${\left[\mathrm{Fe}{\left(\mathrm{CN}\right)}_{6}\right]}^{3-}/{\left[\mathrm{Fe}{\left(\mathrm{CN}\right)}_{6}\right]}^{4-}$ are displayed in Figure 6. All the key measurements, including voltage and current values recorded during three cycles, are presented in Table 3 for both systems. Additionally, average (avg) and standard deviation (SD) calculations provided insights into the performance of the respective devices.

From the analysis of the data in Table 3, multiple considerations were made. First, regarding the oxidation peaks, both systems exhibited similar peak potentials, and the correlation coefficient between the two datasets was 1. The PULSE system showed an average oxidation potential of 170.2 mV, while the benchtop potentiostat recorded 173.4 mV. This corresponded to a difference of 3.2 mV, representing a 1.85% decrease in the PULSE system. The average oxidation currents were comparable: the PULSE system recorded 149.9 μA, and the benchtop potentiostat recorded 154.8 μA, meaning a difference of 4.9 μA, which corresponded to a 3.2% decrease in the PULSE. For reduction peaks, the benchtop potentiostat and the PULSE system averaged 53.3 mV and 54.9 mV, respectively, with a difference of 1.6 mV. Reduction peak currents were alike: the PULSE recorded −145.9 μA, and the benchtop potentiostat recorded −151.8 μA, resulting in a difference of 5.9 μA, which corresponded to a 4% increase in the PULSE. The SD provided insights into data dispersion, and the results suggested that the PULSE offered stable and reliable measurements.

**Figure 7:**
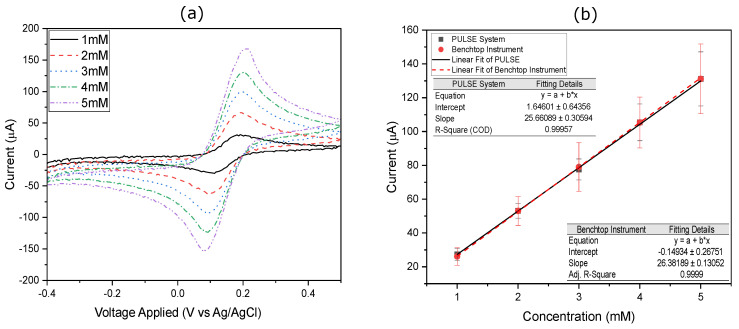
CV results (**a**) using the PULSE, where the potential was swept from −0.5 V to 0.5 V at 50 mV/s with varied concentrations of ${\left[\mathrm{Fe}{\left(\mathrm{CN}\right)}_{6}\right]}^{3-}/{\left[\mathrm{Fe}{\left(\mathrm{CN}\right)}_{6}\right]}^{4-}$ in 10 mM PBS, and (**b**) calibration curves of oxidation peak current against Fe concentrations using the PULSE and the commercial instrument, with linear fits (*n* = 3). Error bars represent standard deviation.

**Figure A1:**
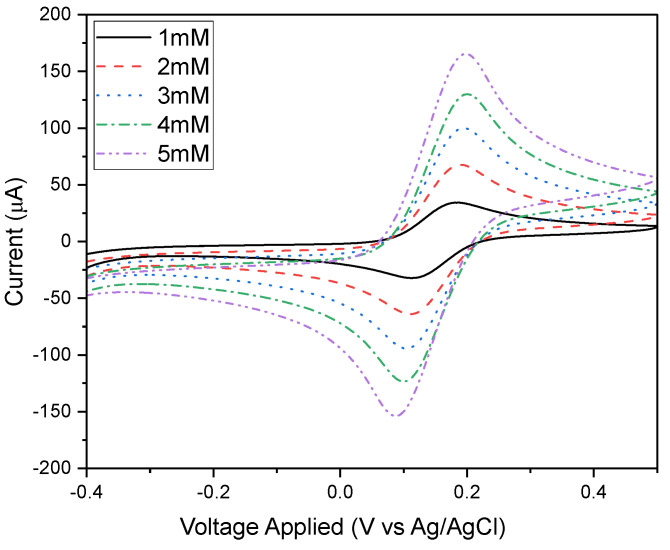
CV results using the benchtop potentiostat, where the potential was swept from −0.5 V to 0.5 V at 50 mV/s with varied concentrations of ${\left[\mathrm{Fe}{\left(\mathrm{CN}\right)}_{6}\right]}^{3-}/{\left[\mathrm{Fe}{\left(\mathrm{CN}\right)}_{6}\right]}^{4-}$ in 10 mM PBS for the first tested sensor.

To further validate the system’s performance, this work tested different concentrations of ${\left[\mathrm{Fe}{\left(\mathrm{CN}\right)}_{6}\right]}^{3-}$/$\mathrm{Fe}{\left(\mathrm{CN}\right)}_{6}{]}^{4-}$ in triplicate using CV measurements. The concentrations ranged from 1 mM to 5 mM at a scan rate of 50 mV/s from −0.5 V to 0.5 V. The results obtained with the PULSE are shown in Figure 7a, while the results using the benchtop potentiostat are displayed in Figure A1. To facilitate comparison between these systems, the peak oxidation currents were analysed and are presented in Figure 7b, where the oxidation peak current is plotted against the solution concentration for both the PULSE and the benchtop instrument calibration curves. For more details, refer to Supplementary Information Table S1.

The PULSE calibration line exhibited a slope of 25.66 μA/mM. The model showed an excellent fit to the data, with an $R^{2}$ value of 0.9996, indicating that 99.96% of the variance in the current can be explained by the linear regression model. Similarly, the linear regression analysis of the dataset obtained from the benchtop potentiostat resulted in a slope of 26.38 μA/mM and an $R^{2}$ of 0.999. A correlation analysis yielded a coefficient of 0.999 between the datasets from both systems. Therefore, it is justified to conclude that the data obtained from the benchtop potentiostat and the PULSE system were highly comparable.

### 3.4. Chronoamperometry

**Figure 8:**
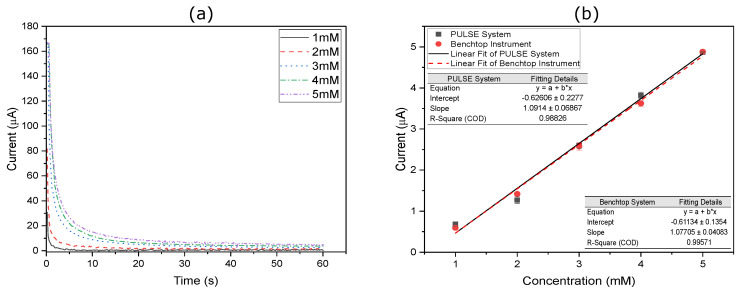
CA results using (**a**) the PULSE, where a constant potential was applied to solutions of varying Fe concentrations for 60 s, and (**b**) represents the calibration curves for the resulting current against the Fe concentrations using the PULSE and the benchtop instrument in triplicate (*n* = 3). Error bars represent the standard deviation.

**Figure A2:**
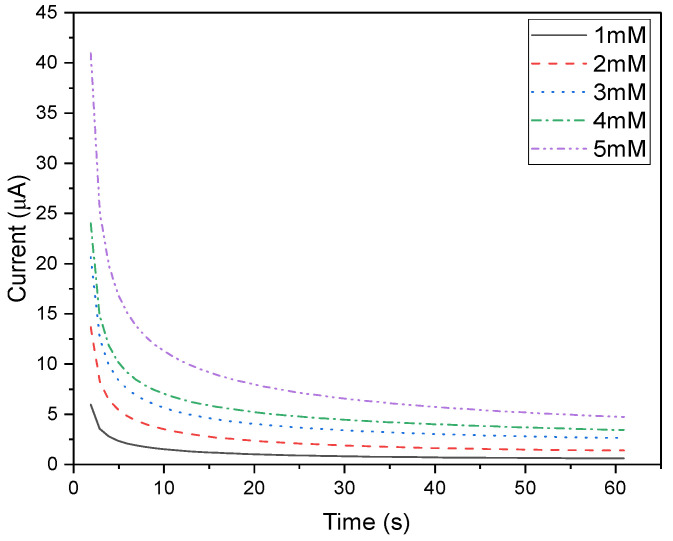
CA results using the benchtop potentiostat where a constant potential was applied to solutions of varying ferri-ferro concentrations for 60 s.

Another method used to validate the performance of the PULSE system was CA. In this technique, the potential was held constant over a 60 s period in the region where the oxidation potential occurs. These measurements were performed in triplicate across multiple ferri-ferro solutions with concentrations ranging from 1 mM to 5 mM. The results obtained using the PULSE system are shown in Figure 8a, while those from the benchtop potentiostat are illustrated in Figure A2. The calibration curves were generated for each system, as shown in Figure 8b. For more details, refer to Supplementary Information Table S2.

The experiments revealed that the PULSE system reached the same rate of change faster, approximately within ∼20 s, compared with the benchtop potentiostat, which required around ∼45 s. The benchtop potentiostat is a more complex instrument that requires prolonged stabilization times. For the PULSE system, the slope was 1.09 μA/mM. The model exhibited an excellent fit to the data, with an $R^{2}$ value of 0.988, indicating that 98.8% of the variance in the current was explained by the linear regression model. In comparison, the benchtop potentiostat showed a slope of 1.08 μA/mM, with an $R^{2}$ value of 0.996. Furthermore, the datasets from both systems exhibited a high correlation, with a coefficient of 0.997, indicating that the performance of the PULSE system closely aligned with that of the benchtop instrument.

### 3.5. pH Measurements

**Figure 9:**
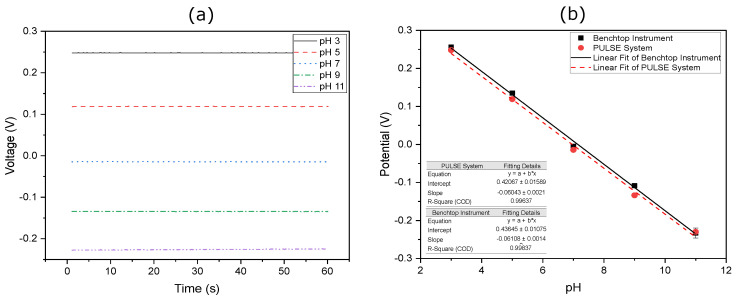
OCP results for various pH solutions over a 60 s period for (**a**) the PULSE and (**b**) resulting calibration plots of potential versus pH for both systems, including linear fits and standard deviation.

**Figure A3:**
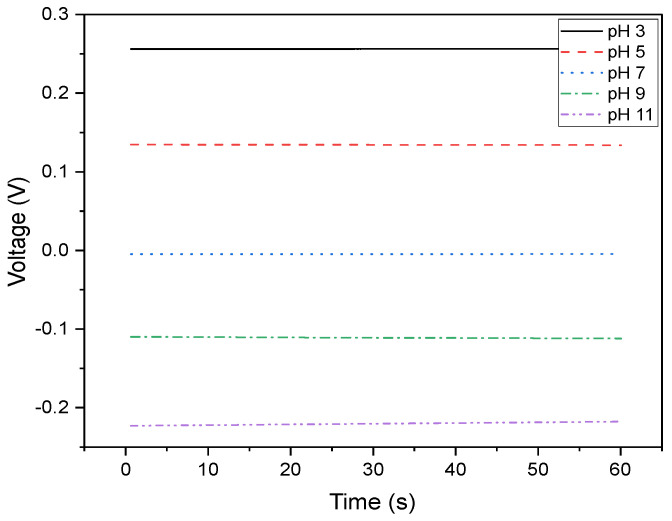
OCP results for various pH buffers over a 60 s period using the benchtop potentiostat.

OCP was used to validate the PULSE system against the benchtop potentiostat. pH was chosen as the target analyte due to its significance in a wide range of applications [24,30]. In the OCP detection of pH, the WE generated a potential as a function of the surrounding solution, which was measured against the constant potential of the RE. Prior to analysis, the WE of the SPE was functionalised with an IrOx sensing layer. PBS solutions with pH values ranging from 3 to 11 were prepared, and the OCP for each pH solution was assessed over a 60 s period in triplicate. The results obtained using the PULSE system are shown in Figure 9a, while those from the benchtop potentiostat are illustrated in Figure A3.

The data indicated that the PULSE system achieved the same rate of change in approximately 2 s, whereas the benchtop potentiostat required around 50 s. This improved speed can be attributed to the sensor stabilization facilitated by the low input bias current, which does not affect the sensor. In contrast, the benchtop potentiostat, being a more complex instrument, required prolonged stabilization times. Furthermore, due to the benchtop instrument’s capabilities to record current at the WE during OCP measurements, currents in the pA range were observed (Equation (7)). This highlighted the importance of incorporating component U7 in the AFE circuitry.

The calibration curve in Figure 9b represents the potential of each solution plotted against the pH value and includes linear regression analyses obtained from both the PULSE system and the benchtop potentiostat. For more details, refer to Supplementary Information Table S3. For the PULSE system, the regression analysis revealed a slope of −60.43 mV/pH with an $R^{2}$ of 0.996, which is close to the ideal −59 mV/pH according to the Nernst equation [31]. This translated into an accuracy of 97.58%. Similarly, for the benchtop potentiostat, the regression analysis showed a slope of −61.08 mV/pH with an $R^{2}$ value of 0.998. Supporting the visible overlap in the datasets, a correlation analysis revealed a coefficient of 0.999 between the datasets from both systems, further emphasizing the accuracy of the PULSE.

### 3.6. Related Work

In this section, the proposed device, PULSE, was compared against other SoTA systems relevant to this study. This comparison focused on their specifications, as detailed in Table 4.

The measurement range highlights the current sensing ability of the PULSE to read output currents in the nA range. Additionally, the potential accuracy was determined to be ±2.03% ± 5.03 mV of the measurement range, and the current accuracy was ±1.88% ± 3.28% of the measurement range, reflecting the maximum deviation between the measured and true values. Furthermore, the proposed device featured a flexible circuit that can be tailored to the specific requirements of the analyte under investigation. While many existing devices focus on voltammetric measurements, including CV, CA, Squarewave Voltammetry (SWV), and Differential Pulse Voltammetry (DPV), the PULSE incorporated a potentiometric circuit for OCP. Furthermore, the PULSE offered a broader voltage range compared with other devices. Due to its versatile operational range, the PULSE system can be used in a wider range of applications than current SoTA systems.

In addition to electrochemical measurements, the PULSE stood out by simultaneously monitoring physical parameters such as temperature and acceleration. Despite being developed as a prototype without specific emphasis on miniaturisation, the PULSE occupied a footprint similar to DStat and is marginally larger than some other devices, yet incorporating many more features. All the devices featured a single sensor connector for the standard 3-electrode configuration. However, [35] developed a dual-channel device with the capability to perform voltammetric measurements using a 3-electrode configuration. In contrast, the PULSE supported simultaneous connections for two sensors in both 3-electrode and 2-electrode configurations.

## 4. Conclusions

This study presented the development of a portable instrument for electrochemical sensing, which was benchmarked against a commercially available benchtop potentiostat. The system was created using modern low-power electronic components, which, when combined with electrochemical measurement techniques, create a compact and efficient solution. The rapid pH detection, within 2 s, by the PULSE system demonstrated the device’s potential for rapid analysis. Combined with its portability and communication features, the PULSE is positioned as a suitable tool for point-of-use applications in multiple scenarios. This work highlighted the device’s operational versatility and customisation potential, allowing tailored configurations to meet specific requirements. Based on the reliable and consistent results obtained from the PULSE, as well as its comparable performance with the benchtop potentiostat, supported by robust correlations (>0.99), it can be concluded that the device presented in this paper is highly suitable and effective for electrochemical measurements. The device’s compact design and communication options via USB and wireless connectivity enhanced its practicality in various environments. Additionally, the voltammetric circuit’s wide operational range and sensitivity to low currents increase its applicability across diverse fields. In practical terms, the PULSE device demonstrated its potential to enhance the speed and quality of measurements by eliminating the need for sample collection, transport, and storage. This approach also reduces associated costs and energy consumption, as it minimises reliance on specialised personnel and equipment.

Furthermore, this study introduced a new portable electrochemical sensing solution with multi-parametric capabilities, holding promise for broader applications in complex fluids and various research domains.

## Figures and Tables

**Table 1:** Operational range of the voltammetric AFE by gain (G) and feedback resistor ($R_{f}$) values.

$R_{f}$ (Ω)	G = 1	G = 2	G = 10	G = 100
1 M	±1.55 μA	±800 nA	±160 nA	±16 nA
9.9 k	±156.57 μA	±80.81 μA	±16.16 μA	±1.62 μA

**Table 2:** Operational ranges of the potentiometric AFE, including WE voltage ($V_{WE2}$) and output voltage ($V_{out2}$) by gain (G).

Signal	G = 1	G = 2	G = 10	G = 100
$V_{WE2}$ (V)	[0; 3.3]	[0.825; 2.475]	[1.485; 1.815]	[1.634; 1.667]
$V_{out2}$ (V)	±1.65	±0.83	±0.165	±16.5 m

**Table 3:** Comparison of oxidation/reduction peak potentials and currents over three cycles for the benchtop instrument and PULSE, including average (Avg) and standard deviation (SD).

System	Benchtop Potentiostat				PULSE System			
Peak	Oxidation		Reduction		Oxidation		Reduction	
Cycle	Pot (mV)	I ( μA)	Pot (mV)	I ( μA)	Pot (mV)	I ( μA)	Pot (mV)	I ( μA)
1	171.8	150.7	51.7	−152.4	171.9	141.6	58.2	−146.6
2	174.3	155.8	54.2	−151.4	171.9	152.5	53.3	−145.3
3	174.3	157.9	54.2	−151.7	166.9	155.5	53.3	−145.9
Avg	173.4	154.8	53.3	−151.8	170.2	149.9	54.9	−145.9
SD	1.18	3.05	1.16	0.44	2.33	5.97	2.33	0.53

**Table 4:** Comparison of portable potentiostats (Meas. = measurements, Comm. = communication).

System	Size (mm)	Meas.	Comm.	Voltage Range (V)	Meas. Range
MiniSat [32]	27 × 20	CA, CV, SWV	Bluetooth	±1.2	±100 μA
DStat [33]	92 × 84	CV, SWV, DPV	USB	±1.5	±600 fA
KAUSTat [34]	50 × 35	CV	Bluetooth	-	±500 μA
[35]	64 × 39	CV, DPV, CA	Bluetooth	-	±10 nA
**PULSE** **(This work)**	**78 × 100**	**Temp., Acc.,** **CV, OCP,** **CA**	**USB,** **433 MHZ**	**±1.65**	±800 **nA ^a^** ±1.6 **V ^b^**

^a^ When voltammetric circuit is set with G = 2 and $R_{f}$ of 1 MΩ. ^b^ When potentiometric circuit is set with G = 1.

**Figure A4:**
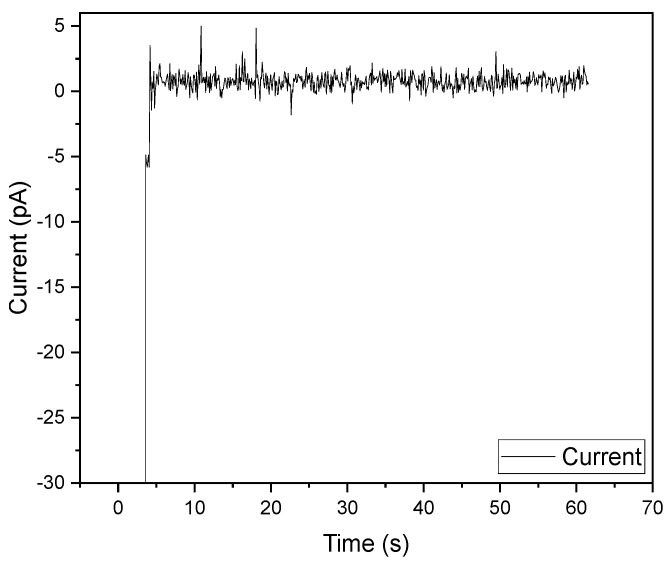
Current at the WE during OCP measurements for pH detection over a 60 s period obtained using the benchtop potentiostat.

## Data Availability

The original contributions presented in this study are included in the article. Further inquiries can be directed to the corresponding authors.

## Supplementary Materials

The following supporting information can be downloaded at: https://www.mdpi.com/article/10.3390/s25030762/s1, Table S1: Calibration of Ferri-Ferro CV peak oxidation values for both systems (*n* = 3); Table S2: Calibration of Ferri-Ferro performing CA in both systems (*n* = 3); Table S3: pH calibration data performing OCP in both systems (*n* = 3).

## Abbreviations

The following abbreviations are used in this manuscript:

ADC	analogue-to-digital converter
AFE	analogue front-end
APP	application
CA	chronoamperometry
CE	counter electrode
CMOS	complementary metal oxide semiconductor
CV	cyclic voltammetry
DAC	digital-to-analogue converter
FSM	finite-state machine
FW	firmware
GUI	graphical user interface
I	current
I2C	inter-integrated circuit
ISM	Industrial, Scientific, and Medical
MCU	Microcontroller Unit
OCP	open circuit potentiometry
PBS	phosphate-buffered saline
PCB	printed circuit board
PPK2	Power Profiler Kit II
PULSE	Portable Unit for Lab-on-Site Electrochemistry
RE	reference electrode
SNR	signal-to-noise ratio
TIA	trans-impedance amplifier
USART	universal asynchronous receiver transmitter
USB	universal serial bus
WE	working electrode
XOR	exclusive OR

## Appendix A. Voltammetric AFE Development

The properties of the chosen control amplifier allowed the user to switch on and off the component. When active, the amplifier drove *C.Sig* into the CE. In contrast, in the inactive state, the amplifier had high impedance, thus preventing any current flow. This supports prolonging the usability of sensors as surface degradation is prevented. When S1 of U2 is enabled, $R_{f}$ is set to 9.9 kΩ, achieved by 1 MΩ ($R_{1}$) in parallel with 10 kΩ ($R_{2}$). The feedback components function as a frequency compensation voltage divider in conjunction with the electrochemical cell, ideally satisfying the equation $R_{f}\times C_{f}=R_{ct}\times C_{dl}$, where $R_{ct}$ represents the charge transfer resistance and $C_{dl}$ represents the double-layer capacitance in the electrochemical cell [36,37].

The gain of the signal is determined by the value of $R_{g}$ of amplifier U4. The resistor value is variable due to the component U3. For example, when switch S1 is on, $R_{G}$ is set to $R_{9}$ (100 kΩ), resulting in a gain of 2 for the INA333. When switch S2 is on, $R_{G}$ becomes $R_{8}$ (11 kΩ), amplifying the input signal by a factor of 10. Similarly, when switch S3 is on, $R_{G}$ is $R_{7}$ (1 kΩ), providing a gain of 100. Lastly, when the switch is connected to S4, no resistor is connected, and the amplifier operates as a unity gain buffer. The output voltage ($V_{out}$) of this circuit can be calculated using Equation (A1), as an adaptation from the components’ data sheet.

(A1) $$V_{out1}=V_{ref}+G\times \left(V_{tia}-V_{Bias1}\right)$$

## Appendix B. Firmware Development

The FW was implemented to control the hardware components, acquire data, process them, and provide user interface functionalities. The chosen MCU is STM32F372R8T6 from STMicroelectronics, capable of handling required tasks with sufficient processing power. The development of the FW was done using the MCU platform STM32 Cube IDE, which provided the necessary resources and interfaces for communication, data processing, and user interaction.

To guarantee the integrity of the measurement, the checksum value was calculated by performing an XOR operation on data bytes, and it was appended to the message before the stop index. The receiver verifies the message integrity by performing the same XOR operation and comparing the calculated checksum with the received one. If the checksums match, it indicates that the message was received without any errors or data corruption [27].

As described previously [38], amperometry focuses on quantifying the steady-state current resulting from an electrochemical reaction, providing quantitative information about the analyte concentration or reaction rate [39]. Once the FSM is started, the voltammetry AFE is enabled, and $V_{ref}$, $V_{Bias1}$, and C.Sig were set to 1.65V using an external DAC (DAC8568 from Texas Instruments). Following a 5 s delay for cell stabilization, C.Sig adjusted to the user-inputted voltage. The MCU started data acquisition, continuously sending acquired data back to the user every 100ms until the stop signal was received. Upon termination, the voltammetric AFE was powered down, and signals reset, halting data acquisition. This approach ensured precise amperometric measurements with voltage control flexibility.

CV involved sweeping the applied potential repetitively to observe redox reactions and determine electrochemical parameters [38,40]. Through the APP, the user can set initial, maximum, and minimum voltages, slope, and number of cycles. Once the start signal was received, the voltammetry AFE was enabled, setting $V_{ref}$, $V_{Bias1}$, and C.Sig to 1.65 V. After a 5 s delay for system stabilization, measurements began, sweeping from initial to maximum and back to minimum voltage at the specified rate for one cycle. The process continued until a stop signal was received or the predetermined cycle number was reached. Upon completion, the AFE was disabled, and signals reset.

Lastly, potentiometry involved measuring the potential between the WE and RE, enabling the investigation of various analytes [38,41]. Using the APP, the user had control over initiating and terminating the measurement. When the MCU received the start signal, the potentiometric AFE was enabled, setting the signals Vref and $V_{Bias2}$ to 1.65 V and initiating the data acquisition. Upon reception of the termination signal, the AFE was disabled, the signals were set to null, and the data acquisition was halted.
